# The study of elastic fibres in oral precancerous and cancerous lesions using Shikata’s modified orcein stain: a retrospective study

**DOI:** 10.3332/ecancer.2024.1798

**Published:** 2024-11-14

**Authors:** Sandhya Tamgadge, Nikita Kamble, Treville Pereira, Avinash Tamgadge, Mayura Chande, Siddharth Acharya

**Affiliations:** 1 Department of Oral and Maxillofacial Pathology and Microbiology, DY Patil University School of Dentistry, Sector 7, Nerul, Navi Mumbai 400706, Maharashtra, India; 2 Department of Public Health Dentistry, DY Patil University School of Dentistry, Nerul Navi Mumbai, Sector 7, Nerul, Navi Mumbai 400706, Maharashtra, India

**Keywords:** elastic fibres, OSCC, OSMF, oral epithelial dysplasias, Shikata’s modified orcein stain, precancer, cancer

## Abstract

**Statement of the problem:**

Oral squamous cell carcinoma (OSCC) is the most common type of oral cancer. During the invasion, tumour cells break through the basement membrane and penetrate the connective tissue to interact with the extracellular matrix. An attempt was made to evaluate the connective tissue changes in different grades of OSCCs, oral submucous fibrosis (OSMF) and Oral Epithelial Dysplasias. Literature related to the evaluation of malignant epithelial cells is vast but very sparse knowledge is available on the role of extracellular stromal fibres on tumour invasion in oral cancer and precancer especially on elastic fibres using Shikata’s Modified Orcein Stain.

**Purpose:**

To analyse the changes in elastic fibres in varying grades of OSCC, OSMF and Oral Epithelial Dysplasias using a special stain, Shikata’s modified orcein stain.

**Materials and method:**

A total of 100 cases were selected as the study group in this retrospective observational study. One section each was cut from 50 samples of varying grades of OSCC and 50 samples of varying grades of OSMF and oral leukoplakia. Ten samples of the control group were taken from the archives of the Department of Oral Pathology. Qualitative and quantitative analysis of elastic fibres was accomplished using set criteria. Spearman’s test was done to evaluate the elastic fibres. Statistically insignificant results were obtained for quantitative and qualitative analysis of elastic fibres.

**Results:**

A change in density in elastic fibres was observed in progression from early to advanced grades of OSCC, OSMF and oral epithelial dysplasia cases. A low expression of elastic fibres in tumour stoma indicates that the protective barrier against tumour progression is deficient, allowing the tumour to progress more easily and resulting in a poorer prognosis.

**Conclusion:**

The elastic fibres undergo a change in density, orientation and packing in the stroma of varying grades of OSCC, OSMF and oral epithelial dysplasia cases. The unique feature of this study lies in the exploration of elastic fibres in OSCC which has not been done so far.

## Introduction

Oral cavity cancer is a major global health concern. Squamous cell carcinoma is a malignant epithelial cell neoplasm exhibiting squamous differentiation as characterised by the formation of keratin and/or the presence of intercellular bridges [1–3]. When a potentially malignant disorder progresses to cancer it undergoes series of histological alterations [4, 5]. The stroma especially stromal fibres plays an important role in the invasion and metastasis [6]. The role of mesenchyme in tumours can indicate the ability of the tumour cells to invade and metastasize [7]. Morphologic detection on haematoxylin and eosin and special stains of connective tissue fibres are cost-effective compared to the molecular markers. Invasion and metastasis can be studied effectively by the surgeon with the use of special stains. There are multiple studies on collagen and its association with metastasis but the association of tumour cells and elastic fibres and its effect on the progression of carcinoma is questionable [8]. Tuxhorn *et al* [9] quoted that the cancer cells can interact specifically with elastin through two elastin-binding proteins and galectin-3. Timar *et al* [10] Sabnis *et al* [11] and Kielty *et al* [12] have suggested that there is a positive correlation between tumour progression and the presence of elastic fibres in the tumour stroma [10–12].

The advantages of the use of orcein for staining elastic fibres in the skin were described in 1891 by Unna [13]. Henwood mentioned that various unpublished studies on elastic tissues failed to demonstrate elastic fibres including the Verhoeffs Van Giesson stain. Therefore, Henwood used Shikata’s orcein technique in his research to demonstrate the hepatitis B surface antigen (HBsAg), copper-associated protein (CAP) and sulfated mucins, which also stained the elastic fibres in the areas of liver fibrosis [14]. In dental literature, Shikata’s modified orcein stain has been used only in lichen sclerosis of the lip [15].

Shikata’s modified orcein stain has been used to study the elastic tissue by Henwood [16, 17].

Verhoeff–van Giesson’s Elastic Stain is used to identify the atrophy of elastic tissue, such as in emphysema, evidence of vascular diseases (arteriosclerosis) and the invasion of tumours into vessels.

In the literature only 2 studies, one by Dineshshankar *et al* [18] and another by John and Murthy [19] have been done to study the morphology of elastic fibres using special stains. They are as follows: Dineshshankar *et al* [18] conducted a comprehensive investigation to evaluate the morphological alterations in elastic fibres, a principal component of connective tissue, across varying grades of oral squamous cell carcinoma (OSCC) and epithelial Oral Epithelial Dysplasias. Their study provided valuable insights into the progressive changes in elastic fibre architecture during oral carcinogenesis [18].

In a related study, John and Murthy [19] examined both collagen and elastic fibres at different stages of OSCC. Their research aimed to correlate these stromal changes with two well-established grading systems: Broder’s and Bryne’s. This comparative approach offered a nuanced understanding of how extracellular matrix (ECM) alterations relate to traditional histopathological grading methods in OSCC [19].

Notably, Katsoulas *et al* [15] employed Shikata’s modified orcein stain in their study of lichen sclerosus of the lip. Their use of this staining technique demonstrates the versatility and potential broader applications of Shikata’s modified orcein stain in visualising elastic fibre changes across various oral lesions [15].

These studies collectively underscore the growing interest and importance of investigating elastic fibre alterations in oral pathologies. They highlight the potential of elastic fibre assessment as a valuable tool in understanding disease progression and potentially aiding in the diagnosis and prognosis of oral lesions. The sparse literature, the lesser number of elastic fibres in lamina propria and the masking effect of overlying inflammatory cells could be the limiting factors in the assessment of elastic fibres in OSCC. This calls for more sensitive labelling systems for assessing elastic fibres (Tables 1 and 2).

This could be is the first study in the literature in which elastic fibres have been evaluated in precancer and cancer using Shikata’s modified orcein stain.

### Aims and objectives

To study the elastic fibres in oral potentially malignant disorders and oral cancerous lesions using Shikata’s modified orcein stain and to correlate with the histological grading using hematoxylin and eosin.

## Materials and method

Ethical approval was obtained from the Institutional Ethics Committee before the commencement of the study. The IREB Reference Number is IREB/2020/OP/01.

The study was conducted over 1 year, from January to December 2021. Histological sections were obtained from the archives of the Department of Oral Pathology and Microbiology. As controls, ten cases of normal oral mucosa were selected, along with a transverse section of the femoral artery as a positive control.

The study group comprised diagnosed cases of OSCC and potentially malignant disorders, including oral submucous fibrosis (OSMF) and oral leukoplakia (oral epithelial dysplasia). The inclusion criteria were histologically confirmed cases with sufficient epithelial and connective tissue. Cases were divided into two categories OSCC: cancerous and precancerous OSCC. The cancerous group OSCC included 50 cases of OSCC, which were further classified into 30 well-differentiated OSCC, 15 moderately differentiated and 5 poorly differentiated cases according to Broder’s classification [20]. The precancerous group included 21 cases of leukoplakia categorised by the severity of epithelial dysplasia (9 mild, 7 moderate and 5 severe) and 29 cases of OSMF were graded as follows: 18 cases in Grade I, 6 in Grade II, 3 in Grade III and 2 in Grade IV, based on the system proposed by Rajendran *et al* [21].

Paraffin-embedded tissue sections were retrieved from the archives for further analysis. A single section was prepared from each paraffin block and stained using Shikata’s modified orcein stain, following the technique described by Henwood [14] to assess the elastic fibre component. The elastic fibres were analysed at magnifications of 4×, 10× and 40× for their density, grouping patterns, staining intensity and orientation relative to the overlying epithelium and tumour islands using a Leica research microscope (Model No. DM1000 LED, Leica Microsystems GmbH, Ernst-Leitz-Straße 17–37, 35578 Wetzlar, Germany). Photomicrographs were captured for documentation purposes.

The density of elastic fibres was categorised as scanty, moderate or abundant. Morphological patterns were classified as long, thin, straight fibres present singly or short, thick, wavy fibres present in clusters. The staining intensity, the orientation of elastic fibres to the overlying epithelium and their orientation to tumour islands were carefully evaluated [22].

All histological slides were reviewed independently by two pathologists who were blinded to the patients’ clinical details. In cases of discrepancy, a consensus was reached using a multi-headed microscope. A minimum of four fields of view at 200× magnification were selected for analysis, and digital images were captured for further evaluation. Statistical analysis of elastic fibre parameters across various grades of OSCC, OSMF and oral leukoplakia was performed using Spearman’s test.

## Results

Under microscopic examination, the elastic fibres were observed as delicate, undulating structures. These fibres were predominantly located within the connective tissue matrix, forming an intricate network throughout the examined tissue sections. Their distinctive wavy appearance and distribution pattern were characteristic features that aided in their identification and analysis.

### Colour of elastic fibres

The elastic fibres stained a deep brown to dark purple colour in the tissue sections, providing a distinct contrast against the background tissue, which stained a lighter shade. This coloration facilitated the clear identification and assessment of the elastic fibres within the oral precancerous and cancerous lesions and positive control sample that is artery wall.

Various morphologic patterns of elastic fibres were observed under the Leica microscope (Tables 3–6).

### Control group

**Figure 1.**
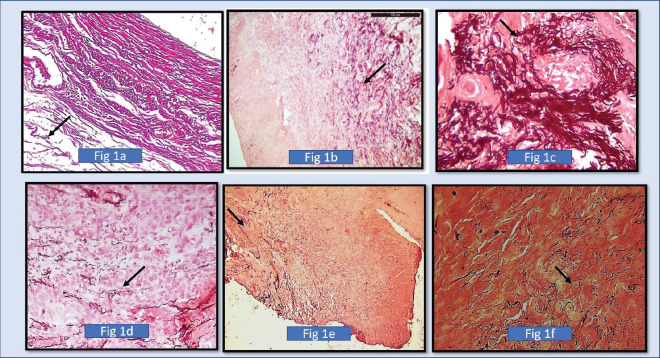
(a): Photomicrograph shows elastic fibres using SHIKATA’S MODIFIED ORCEIN STAIN (dark brown to black coloured stain). In the positive control group in the wall of the blood vessel, (b): Photomicrograph shows fragmented fibres (Morphology). In 10× magnification in normal oral mucous membrane using SHIKATA’S MODIFIED ORCEIN STAIN, (c): Shows photomicrograph of 40× magnification showing short, thick and wavy elastic fibres clumping of elastic fibres in well differentiated squamous cell carcinoma around tumour islands (SHIKATA’S MODIFIED ORCEIN STAIN), (d): Shows photomicrograph of moderately differentiated squamous cell carcinoma showing less density of elastic fibres (4× Magnification) (SHIKATA’S MODIFIED ORCEIN STAIN), (e): Shows photomicrograph of moderately differentiated squamous cell carcinoma showing less density of elastic fibres. (4× magnification) (SHIKATA’S MODIFIED ORCEIN STAIN), and (f): Shows photomicrograph of short, fragmented, thin straight elastic fibres in poorly differentiated squamous cell carcinoma. (40× Magnification) (SHIKATA’S MODIFIED ORCEIN STAIN).

The elastic fibres in the control group showed normal long fibres, clumped fibres and fragmented fibres, but long normal-looking fibres were in the majority (Figure 1).

### Study group

When the density of the elastic fibres was analysed, abundant to moderate fibres were noted in well differentiated OSCC, moderately differentiated OSCC, OSMF grade 1 and 2 and scanty fibres were noted in poorly differentiated OSCC, OSMF grade 3 and OSMF grade 4; whereas in oral epithelial dysplasias the density was moderate to abundant in mild and moderate oral epithelial dysplasiascases, and scanty in severe oral epithelial dysplasias.

When morphology and pattern of grouping as evaluated in both the subgroups of study groups, the milder histopathological grades showed a combination of short, thick and wavy, whereas the higher grades showed short, thin and straight fibres. The elastic fibres were present in bunches and singly irrespective of the grades in a few cases in OSCC. Elastic fibres were fragmented or thin clusters/bunches in higher grades of the Oral Potentially Malignant disorders and in their initial grades were mostly present in bunches.

The evaluation of morphology noted short, thick, wavy fibres in both well-differentiated OSCC, Moderately differentiated OSCC, OSMF grade 1 and OSMF grade 2 and in mild oral epithelial dysplasias and moderate oral epithelial dysplasias; whereas as the grades progressed, short, thin, straight fibres were seen in poorly differentiated OSCC, OSMF grade 3, OSMF grade 4 and in severe Oral Epithelial Dysplasias. As grade progresses fibres became long/short, thin and straight (poorly differentiated OSCC and severe oral epithelial dysplasias).

The grouping pattern of the elastic fibres were evaluated as present singly, in bunches and fragmented/clustered. The well-differentiated OSCC, OSMF GRADE 1 and mild oral epithelial dysplasia showed elastic fibres singly present and in bunches. Elastic fibres showed fragmentation and were seen as short fibres in thin clusters in most of the higher grades of the cases taken in precancerous lesions/disorders group.

The findings for the staining intensity for precancerous and cancerous lesions with various grades were inconsistent.

**Figure 2.**
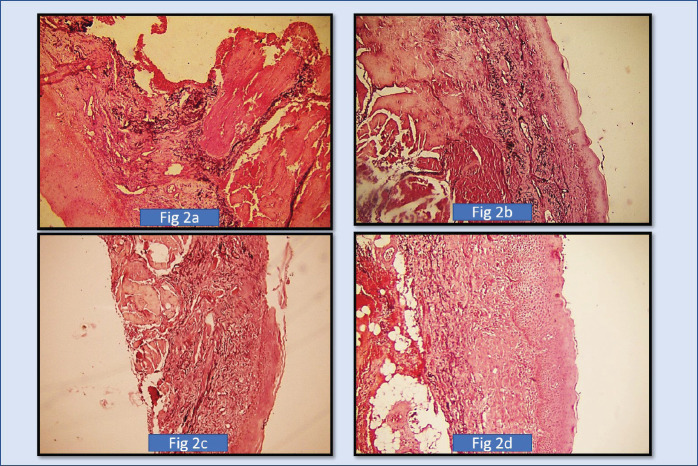
(a): Shows photomicrograph of short, thick, wavy elastic, fibres in OSMF Grade 1 (4× Magnification) (SHIKATA’S MODIFIED ORCEIN STAIN, (b): Shows photomicrograph of short, thick, wavy elastic fibres in OSMF Grade 2. (4× Magnification) (SHIKATA’S MODIFIED ORCEIN STAIN), (c): Shows photomicrograph of OSMF short, thin, straight, elastic fibres in OSMF Grade 3. (4× Magnification) (SHIKATA’S MODIFIED ORCEIN STAIN), (d): Shows photomicrograph of short, thin, straight, fragmented elastic fibres in OSMF Grade 4. (10× Magnification (SHIKATA’S MODIFIED ORCEIN STAIN).

**Figure 3.**
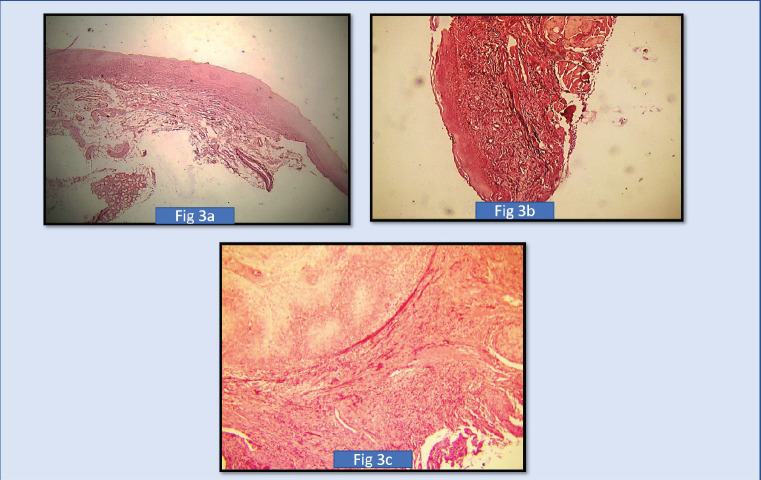
(a): Shows photomicrograph of short thick wavy elastic fibres, in mild oral epithelial dysplasias (4× Magnification) (SHIKATA’S Modified Orcein Stain), (b): Shows photomicrograph of moderate oral epithelial dysplasiasas short, thick wavy elastic fibres (4× Magnification) (SHIKATA’S MODIFIED ORCEIN STAIN). (c): Shows photomicrograph of long, thin, short fibres in severe oral epithelial dysplasias (4× Magnification) (SHIKATA’S MODIFIED ORCEIN STAIN).

The orientation of elastic fibres to epithelium and orientation to tumour islands is parallel in the initial grades of OSCC. Few cases of initial grades showed haphazard/perpendicular arrangement of elastic fibres. The orientation of elastic fibres to epithelium and orientation to tumour islands is haphazard and showed no significant arrangement across various grades of potentially malignant disorders/lesions (Figures 1a–e, 2a–d and 3a–c).

**Figure 4.**
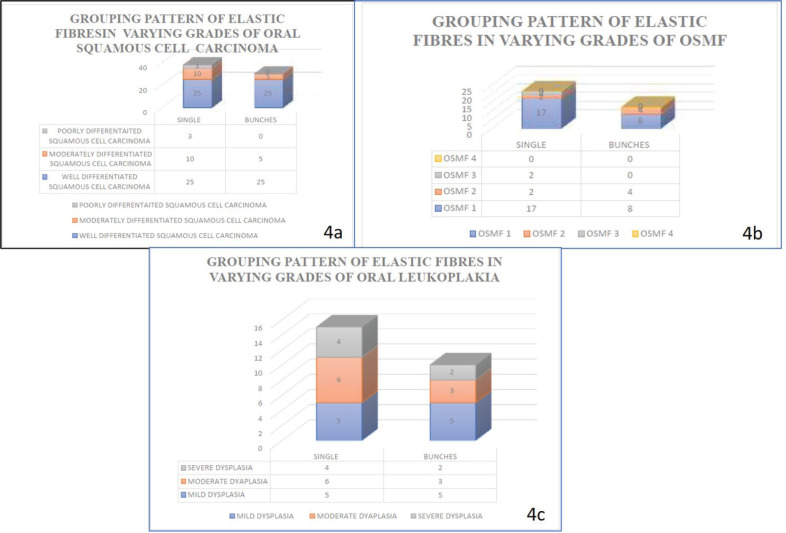
Graph shows the morphology of elastic fibres in various study groups.

**Figure 5.**
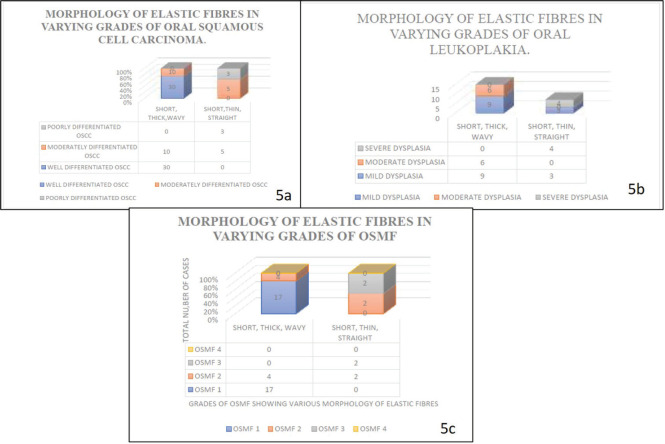
Graph shows grouping pattern of elastic fibres in various study groups.

**Figure 6.**
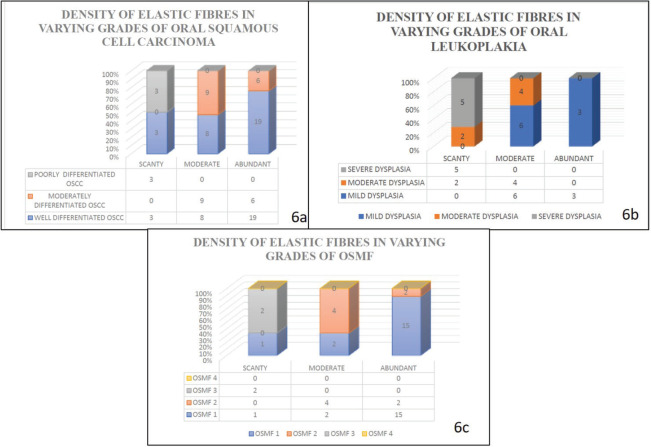
Graph shows the density of elastic fibres in various study groups.

Our study revealed that the elastic fibres were predominantly seen in the initial stages of cancer and potentially malignant disorders/lesions. Scanty or no fibres were seen in the advanced stages of cancer and potentially malignant disorders/lesions. The sparse literature, the lesser number of elastic fibres in lamina propria and the masking effect of overlying inflammatory cells could be the limiting factors in the assessment of elastic fibres in OSCC. This calls for more sensitive labelling systems for assessing elastic fibres (Figures 4–6).

When the density of elastic fibres (abundant, moderate and scanty), were correlated within the OSCC group, the statistical analysis was significant in all earlier grades as compared to advanced grades, with a significant *p* value < 0.05 (*p* = 0.002) The statistical analysis showed *p* value less than 0.05 (*p* = 0.002) which is significant with Spearman’s ratio.

When the staining intensity (strong, moderate and weak) and the orientation of elastic fibres to overlying epithelium (parallel, perpendicular/haphazard) were correlated within the OSCC group, the statistical analysis was significant in all earlier grades as compared to advanced grades (staining intensity (*p* = 0.025) and (*p* = 0.00) for orientation of elastic fibres to overlying epithelium with the Spearman’s ratio).

The statistical analysis for the morphology of elastic fibres in OSCC cases (*p* > 0.05)] (*p* = 0.183) (short, thick, wavy, long, thin and straight) and pattern of grouping (present singly, in bunches or clustered/fragmented) *p* value >0.05 (*p* = 0.869) was not significant with Spearman’s ratio (Tables 4–6) Figures 4–6.

Most of the cases of the well-differentiated OSCC showed a parallel arrangement of elastic fibres around some tumour islands and to overlying epithelium whereas in some cases of well-differentiated OSCC and higher grades showed haphazard and absence of elastic fibres except for a few cases. The staining intensity was milder in the initial grades of the OSCC and was moderate to strong in higher grades in OSCC except for a few cases.

The statistical analysis for the pattern of grouping (present singly, in bunches or clustered/fragmented) of elastic fibres was significant with the Spearman’s ratio *p* value <0.05 (*p* = 0.036) for oral potentially malignant lesions/disorders group. The higher grades of the oral potentially malignant disorders group showed elastic fibres in fragmented/clustered form and in thin bunches. The milder grades of the oral potentially malignant disorder/lesions group showed elastic fibres mostly in thick bunches and presented as singly.

The statistical analysis of elastic fibres for staining intensity (strong, moderate and weak) in Oral potentially malignant lesions/disorders group was not significant with Spearman’s ratio *p* > 0.05 (*p* = 0.46) The statistical analysis for density of elastic fibres in oral potentially malignant lesions/disorders group was not significant *p* > 0.05 (*p* =0.907).

The statistical analysis for the morphological patterns of elastic fibres in oral potentially malignant lesions/disorders group (OSMF and ORAL LEUKOPLAKIA) was not significant *p* > 0.05 (*p* = 0.059) no. 8). The *p* value was more than 0.05 for all the morphological parameters of elastic fibres (short, thick, wavy, straight, long, thick and thin) and orientation to overlying epithelium (parallel, perpendicular and haphazard). The *p* value >0.05 for the orientation of elastic fibres to overlying epithelium (parallel, perpendicular or haphazard) (*p* = 0.734) for the Oral Potentially Malignant lesions group is not significant. Out of all parameters for elastic fibres–density, morphology, staining intensity and orientation of elastic fibres to overlying epithelium, the statistical analysis shows that the *p*-value was more than 0.05 which is not significant in oral potentially malignant disorders/lesions.

## Discussion

In this study, we employed Shikata’s modified orcein stain due to its cost-effectiveness, ease of use for novice researchers and the ready availability of required reagents. This staining method provides an accessible and efficient means of visualising elastic fibres in tissue samples.

The literature has documented therapeutic approaches that incorporate elastic molecules to enhance the healing process. These findings underscore the potential significance of elastic fibres in tissue repair and regeneration [22].

Tosios *et al* [23] in their investigation of elastofibromatous changes and hyperelastosis in oral mucosa, observed that the connective tissue exhibited chronic and subacute inflammation, accompanied by increased vascularity and fibrosis. They noted a progressive loss of tissue elasticity correlating with the advancing grades of the lesion. Our study of OSMF revealed similar patterns across various grades of the condition [23].

These observations collectively highlight the importance of elastic fibres in maintaining tissue integrity and function, as well as their potential role in the pathogenesis and progression of oral mucosal disorders. The alterations in elastic fibre distribution and morphology may serve as valuable indicators of disease severity and progression in conditions such as OSMF.

Nuclear pleomorphism and perineural invasion have been identified as crucial parameters in assessing cancer aggressiveness, as demonstrated by Jamadar *et al* [24] These findings underscore the importance of evaluating such areas for elastic fibre distribution and morphology in future studies. The relationship between these histopathological features and elastic fibre alterations could provide valuable insights into the mechanisms of tumour progression and invasion.

Kardam *et al* [25] conducted a comprehensive analysis of stromal fibrosis in OSCC utilising picrosirius red and Verhoeff’s Van Gieson stains. Their study revealed a notable shift in collagen fibre characteristics as the tumour progressed from well-differentiated to poorly differentiated OSCC. Specifically, they observed a predominance of thin collagen fibres exhibiting a greenish–yellow hue, while thick fibres displayed a diverse colour spectrum as the tumour grade advanced. The collagen fibres were found to be loosely packed and haphazardly arranged, suggesting a significant disruption of the ECM architecture. Our study followed similar guidelines but for elastic fibres using Shikata’s Modified Orcein Stain. The quantitative analysis of collagen and qualitative assessment of elastic fibres in Kardam *et al* [25]’s study yielded statistically significant results, highlighting the potential of these stromal changes as markers of tumour progression. These findings were corroborated by John and Murthy [19] and align with our observations in both precancerous and cancerous groups.

The intricate relationship between chronic inflammation and cancer development has been the subject of intense research in recent years. Tlsty and Coussens [26] provided compelling evidence that chronic inflammatory disorders significantly enhance the overall risk for cancer development. Our study builds upon this foundation, offering novel insights into the role of elastic fibres in this process.

We observed a striking absence of elastic fibres in areas of inflammation, particularly in advanced grades of the study group. This finding suggests a potential mechanistic link between the inflammatory microenvironment and the degradation of elastic fibres. The correlation between inflammation and elastic fibre alteration that we identified may represent a critical step in the progression from chronic inflammation to neoplastic transformation.

The ECM plays a pivotal role in regulating tumour behaviour and disease progression. As noted in previous studies, inappropriate synthesis or degradation of any ECM component can profoundly alter cell physiology [27]. Our research provides compelling evidence supporting this concept, demonstrating both qualitative and quantitative alterations in elastic fibres across different stages of disease progression.

Specifically, we observed progressive disorganisation and fragmentation of elastic fibres correlating with advancing disease stages. In the early stages, elastic fibres appeared relatively intact, forming an organised network within the ECM. However, as the disease progressed, we noted a marked decrease in fibre density, increased fragmentation and a loss of the normal architectural arrangement.

Quantitative analysis revealed a statistically significant reduction in elastic fibre content in advanced stages of disease compared to early stages and normal tissue. This quantitative change was accompanied by qualitative alterations, including changes in fibre thickness, continuity and spatial distribution. These observations suggest that elastic fibre degradation is not merely a consequence of disease progression but may actively contribute to the creation of a permissive microenvironment for tumour growth and invasion.

The absence of elastic fibres in areas of intense inflammation in advanced disease stages is particularly intriguing. This finding implies that inflammatory processes may directly or indirectly promote elastic fibre degradation, possibly through the action of inflammatory cell-derived proteases or by altering the expression of elastic fibre-associated proteins.

Elastic fibres play a crucial role in shaping the tumour microenvironment (TME). In the initial phases of tumour development, these fibres are abundant, contributing to the structural framework of the tissue [28–30]. However, as cancer progresses, the quantity of elastic fibres significantly decreases. In advanced cancers, the tumour stroma becomes progressively stiffened, a phenomenon that serves to protect the tumour from host defence mechanisms [31–33]. This stiffening is often associated with increased collagen deposition and other ECM modifications, leading to a more rigid microenvironment that promotes tumour survival and invasion. Matrix degradation can additionally promote malignant progression and metastasis as suggested by Erler and Weaver *et al* [31, 34]. These findings have significant implications for our understanding of disease progression in oral potentially malignant disorders and oral cancer. The alterations in elastic fibres could serve as a valuable biomarker for disease staging and prognosis. Moreover, targeting the processes involved in elastic fibre degradation or promoting elastic fibre preservation could represent a novel therapeutic approach.

The alterations in elastic fibres observed in our study align with several hallmarks of cancer as described by Hanahan and Weinberg [35–38]. Elastic fibre degradation may contribute to ‘sustaining proliferative signalling’ through altered mechanotransduction as suggested by Pickup *et al* [39], ‘activating invasion and metastasis’ by compromising tissue integrity as mentioned by Lu *et al* [34], and ‘tumour-promoting inflammation’ via its correlation with inflammatory processes as stated by Tlsty and Coussens and various authors. *et al* [26, 34, 38, 40]. The disruption of elastic fibre architecture could also play a role in ‘inducing angiogenesis’ [41] and These findings suggest that elastic fibre alterations are not merely a consequence of cancer progression but actively participate in multiple cancer hallmarks, highlighting their significance in tumour biology and potential as therapeutic targets.

They undergo dynamic changes in the accompanying tumour and results in desmoplasia and is associated with the recruitment of inflammatory cells and activation of angiogenic programmes [16].

The TME plays a crucial role in the progression of oral precancerous and cancerous lesions, with cancer-associated fibroblasts (CAFs) emerging as key players in this process. CAFs are a primary source of matrix metalloproteinases (MMPs), enzymes known to degrade ECM components, including elastic fibres according to Monteran and Erez [42]. The degradation of elastic fibres by MMPs may contribute to the altered tissue architecture observed in our study using Shikata’s modified orcein stain.

While CAFs, often identified by markers such as fibroblast activation protein α, have been implicated in promoting tumour growth, metastasis and angiogenesis across various solid tumours [36, 43], our study focuses on the direct visualisation of elastic fibres. The changes in elastic fibre distribution and morphology may reflect the degree of stromal activation and remodelling in oral precancerous and cancerous lesions.

Khalid *et al* [44] demonstrated an increased expression of myofibroblasts, a CAF subtype, in precancerous and cancerous oral lesions, with higher expression correlating with advanced grades of neoplasia. This finding complements our investigation of elastic fibres, as both may serve as indicators of disease progression and TME alterations.

Neoplasms undergoing malignant transformation exhibit increased architectural disarray at the invasive front of the tumour mass. At this site, there is enhanced production of matrix-remodelling enzymes and synthesis of various ECM components, particularly Type I collagen. MMPs, produced by CAFs and inflammatory cells, degrade key ECM structural components, including both collagens and elastic fibres, facilitating neoplastic progression as stated by Dineshshankar *et al* [18] Similar findings were observed in our study where advanced stages of potentially malignant and cancerous lesions, where elastic fibres appeared thin, short and fragmented.

Henwood [14] used the modified Shikats’s orcein staining, an old-established method for the demonstration of elastic fibres, thus providing a simple way of distinguishing collapse from fibrosis. It can be used for the demonstration of elastic fibres, HBsAg [14] and CAP in liver biopsies [16].

The density of elastic fibres was abundantly seen in the initial grades of cancerous and potentially malignant disorders group. Short, thick, wavy fibres were mostly observed in the initial grades of OSCC and potentially malignant disorders groups (ORAL LEUKOPLAKIA and OSMF) whereas long, thin, wavy with an insignificant amount of straight elastic fibres were seen in the higher grades of the cases taken for evaluation. Similar findings were seen by Dineshshankar *et al* [18] and John and Murthy [19] where he used Verhoef’s Van Geisson stain and Picrosirius red stain There was a significantly increased amount of fragmented/short/thin elastic fibres in poorly differentiated/Grade 3 and 4 OSMF/severe Oral Epithelial Dysplasias when compared to normal mucosa. In our study, the findings were inconsistent due to unequal numbers of cases in varying grades. Staining intensity too was inconsistent which might be due to minor changes during each lot of staining. Therefore, immunofluorescence is recommended over histochemical staining because the stain fades over a period of time.

There are various methods to visualise elastic fibres such as special stains, scanning electron microscopy and fluorescent microscopy. Since scanning electron microscopy and fluorescent microscopy are very expensive, the histochemical staining procedure is very effective and reliable [45].

Once prepared by mixing various reagents, the solution has an extended shelf life and can be stored at room temperature. Therefore, Shikata’s modified orcein stain is highly recommended. The OSMF cases in this study were clinically graded as OSMF Grade I, II, III and IV, based on the mouth opening criteria provided by Rajendran and Sivapathasundharam *et al* [46]. Given that OSMF exhibits extensive connective tissue fibrosis, this study also evaluated stromal elastic fibres in addition to collagen. In terms of the orientation of elastic fibres relative to the overlying epithelium and around tumour islands, the fibres were haphazardly arranged, without any significant parallel or perpendicular pattern.

Some tables showed fewer than 50 cases due to the exclusion of samples where specific parameters could not be accurately assessed. This may be due to factors such as inadequate tissue preservation, staining artefacts or absence of evaluable elastic fibres in some sections. To maintain the integrity of the analysis, only cases with clear, assessable features for each specific parameter were included in the respective tables. This approach ensures that the data presented is of high quality and reliability, even if it results in a reduced sample size for certain analyses. Future studies with larger sample sizes may help to further validate these findings.

## Highlight

This would be the first study on the Description of Shikata’s modified orcein stain and its advantages in highlighting elastic fibres in OSCC and oral precancer.

The manuscript provides a valuable contribution to the scientific community by shedding light on the role of elastic fibres in the oral mucosa during the progression of precancerous conditions to cancer. The use of Shikata’s modified orcein stain enhances the precision of the analysis, making the findings particularly relevant for clinicians and researchers working in the field of oral pathology.

## Recommendations and future scope

Future studies can be carried out with Shikata’s modified orcein stain to study elastic tissue in the hard tissue sections.

Future studies should aim to elucidate the molecular mechanisms underlying the observed elastic fibre alterations, particularly in the context of inflammation. Investigation of the specific proteases involved in elastic fibre degradation and the signalling pathways regulating their expression could provide valuable targets for intervention. Additionally, longitudinal studies correlating elastic fibre changes with disease outcomes could further establish their prognostic value.

## Conclusion

In conclusion, this retrospective study utilising Shikata’s modified orcein stain reveals significant insights into the role of elastic fibres in oral precancerous and cancerous lesions. The density, morphology and pattern of elastic fibres correlated significantly with the grades of OSMF, oral leukoplakia and OSCC, while other parameters showed no significant correlation. In OSCC, elastic fibres exhibited haphazard arrangements and fragmentation, indicating extensive remodelling of the ECM. Moreover, the aggressiveness of tumours was linked to stromal changes, with degradation of elastic fibres at the advancing front. These qualitative and quantitative alterations suggest that elastic fibres could serve as adjunct parameters in histological grading. Ultimately, we conclude that the abundance of elastic fibres decreases as the grade of cancer and potentially malignant disorders increases, emphasising their potential role as biomarkers in tumorigenesis.

## Conflicts of interest

The authors declare no conflicts of interest.

## Funding

This research did not receive any specific grant from funding agencies in the public, commercial or not-for-profit sectors.

## Figures and Tables

**Table 1.** Shows timeline of various elastic stains.

Sr. No	Year	Author(s)	Stain name/Method
1.	1890	Unna [13]	Orcein stain
2.	1890	Unna [47]	Orcein method
3.	1898	Weigert and Workman [48]	Weigert's stain (Weigert’s aldehyde stain)
4.	1908	Verhoeff [49], Gieson [50]	Verhoeff or Verhoeff-van Gieson Elastic Tissue Stain
5.	1923	Russell [51]	Movat pentachrome stain
6.	1929	Masson [52]	Masson’s trichrome stain
7.	1950	Gömöri [53]	Aldehyde Fuchsin stain (Gomori's aldehyde-fuchsin stain)
8.	1974	Shikata et al [54]	Shikata’s modified orcein stain
9.	1975	Verhoeff [55]	Iron hematoxylin method
10.	1976	Churukian and Schenk [56]	Alcian blue and Verhoeff’s or iron gallein stain
11.	1978	Krobock et al [57]	Giemsa as a counterstain with orcein
12.	1979	Meloan SN, Puchtler HA	Resorcin-fuchsin methods associated with Weigert
13.	1986	Singh and Gorton [58]	Orcein stain and Alcian blue
14.	1986	Chalvardijian and Lee [59]	Tetrachrome stain (using hematoxylin, orcein, phloxine, and saffron)
15.	2000	Steven et al [60]	Orcein-picroindigocarmine (a new multiple stain)

**Table 2.** Various combinations of orcein stain and other stains used for different tissue components.

S.no	Year	Author	Work done
1.	1890	Unna [13]	Discovered orcein stain.
2.	1974	Shikata [61]	Detected cytoplasmic inclusion bodies and hepatitis B antigens using modified orcein stain.
3.	1976	Salaspuro and Sipponen [62]	Applied Shikata’s modified orcein stain in studies.
4.	1977	Nakamura and Kanai [63]	Investigated orcein staining at the electron microscope level.
5.	1980	Scheuer and Maggi [64]	Studied the collapse of liver parenchyma, as seen in acute hepatitis and bridging necrosis.
6.	1981	Henwood [14]	Researched orceinophilic mucin.
7.	1981	Shousha and Boxer [65]	Investigated acidophilic mucin.
8.	1983	Henwood, [14]	Studied Shikata’s stain for sulfated mucins.
9.	1983	Dawson et al [66]	Used HB8 immunolabelling for pathological conditions, confirming findings with a standard elastic tissue stain.
10.	1983	Henwood [16]	Developed Shikata’s modified orcein technique for demonstrating elastic tissue, hepatitis B surface antigen (HBsAg), copper-associated protein (CAP), and sulfated mucins.
11.	1984	Singh and Gorton [58]	Developed a new orcein-alcian blue staining method to demonstrate sulfated and sialomucins in gastrointestinal epithelium, comparing it with the high iron diamine-alcian blue technique.
12.	1984	Seuba [61]	Compared various staining methods for HBsAg, highlighting the effectiveness of Shikata’s orcein method and its comparison to immunofluorescence and Victoria blue methods.
13.	1986	Verhoeff [67]	Developed Verhoeff’s iron hematoxylin stain for elastic fibers.
14.	1987	Kim [68]	Demonstrated HBsAg-positive cells using orcein, while addressing its limitations, and introduced Victoria blue staining for clearer HBsAg detection.
15.	1989	Singh and Gorton [58]	Used Shikata’s modified orcein stain with alcian blue to demonstrate mucins and sialomucins.
16.	2017	Henwood [14]	Demonstrated four parameters using Shikata’s modified orcein stain: CAP, HBsAg, elastic tissue, and gastrointestinal mucins.

**Table 3.** Density of elastic fibre in cancer versus precancer.

Count						
		Precancer				Total
		No fibre	Scanty	Moderate	Abundant	
OSCC	No fibre	0	2	0	0	2
	Scanty	3	0	1	2	6
	Moderate	0	2	8	7	17
	Abundant	1	6	7	11	25
Total		4	10	16	20	50
**Chi-square tests**						
	Value	df	Asymptotic significance (2-sided)			
Pearson chi-square	27.010^a^	9	0.001			

There is a significant association between density of elastic fibre in cancer and precancer at *p* ≤ 0.05

**Table 4.** Pattern of grouping of elastic fibres in cancer versus in precancer.

Count										
		Precancer								Total
		Cluster	Bunch		Single	Cluster bunch	Cluster single	Bunch single	Cluster bunch single	
OSCC	Cluster	6	3		1	0	0	0	3	13
	bunch	5	3		2	5	0	1	0	16
	Cluster bunch	1	0		2	0	0	0	0	3
	Cluster single	1	1		1	1	0	0	1	5
	Bunch single	0	0		0	0	1	1	1	3
Total		13	7		6	6	1	2	5	40
Chi-square tests										
	Value	df		Asymptotic significance (2-sided)						
Pearson chi-square	38.489^a^	24		0.031						

There is a significant association between pattern of grouping cancer and pattern of grouping precancer at *p* ≤ 0.05

**Table 5.** Staining intensity of elastic fibres in cancer versus in precancer.

Count								
	Precancer							Total
		Weak	Moderate	Strong	Weak moderate	Weak strong	Moderate strong	
OSCC	Weak	1	4	8	1	1	0	15
	Moderate	0	3	6	4	3	0	16
	Strong	1	0	5	0	2	1	9
	Weak Moderate	0	0	0	1	0	0	1
Total		2	7	19	6	6	1	41
**Chi-square tests**								
	Value	df	Asymptotic Significance (2-sided)					
Pearson chi-square	18.257^a^	15	0.249					

There is a no significant association between staining intensity in cancer and staining intensity

**Table 6.** Orientation of elastic fibres in cancer versus in precancer.

Count				
	Precancer			Total
		Parallel to epithelium	Haphazard	
OSCC	Parallel to epithelium	8	15	23
	Haphazard	6	21	27
Total		14	36	50
Chi-square tests				
	Value	df	Asymptotic significance (2-sided)	
Pearson chi-square	0.972^a^	1	0.324	
N of valid cases	50			

There is no significant association between orientation cancer and orientation precancer at *p* ≤ 0.05

## References

[1] Pindborg JJ, Reichart PA, Smith CJ (1997). Histological Typing of Cancer and Precancer of the Oral Mucosa.

[2] Thompson LDR (2017). Update from the 4th edition of the World Health Organisation classification of head and neck tumours: tumours of the ear. Head Neck Pathol.

[3] Ariyoshi Y, Shimahara M, Omura K (2008). Epidemiological study of malignant tumours in the oral and maxillofacial region: survey of member institutions of the Japanese Society of Oral and Maxillofacial Surgeons, 2002. Int J Clin Oncol.

[4] William G (2006). Shafer, Hine L. Shafer ’ s Text Book of Oral Pathology.

[5] Tamgadge SA, Ganvir SM, Hazarey VK (2012). Oral leukoplakia: transmission electron microscopic correlation with clinical types and light microscopy. Dent Res J (Isfahan).

[6] Langley RR, Fidler IJ (2011). The seed and soil hypothesis revisited-the role of tumour-stroma interactions in metastasis to different organs. Int J Cancer.

[7] van den Hooff A (1988). Stromal involvement in malignant growth. Adv Cancer Res.

[8] Chandni S, Tamgadge S, Tamgadge A (2024). Tumour microenvironment in oral squamous cell carcinoma: special stains and scanning electron microscopic study. J Microsc Ultrastruct.

[9] Tuxhorn JA, Ayala GE, Rowley DR (2001). Reactive stroma in prostate cancer progression. J Urol.

[10] Timar J, Lapis K, Fulop T (1991). Interaction between elastin and tumour cell lines with different metastatic potential; in vitro andin vivo studies. J Cancer Res Clin Oncol.

[11] Sabnis SL, Kulkarni MM, Shinde S (2020). Morphological changes of extracellular matrix in different histopathological grades of oral squamous cell carcinoma. Ann Rom Soc Cell Biol.

[12] Kielty CM (2006). Elastic fibres in health and disease. Expert Rev Mol Med.

[13] Unna P (1899). Unna’s Dermatologische Preisaufgabe.

[14] Henwood T (1983). Shikata’s Orcein stain – a routine stain for liver biopsies. Aust J Med Lab Sci.

[15] Katsoulas N, Prodromidis G, Nikitakis NG (2018). Lichen sclerosus of the upper lip: report of a case, utilising Shikata’s modified orcein stain, and review of the literature. J Oral Maxillofac Res.

[16] Henwood A (2003). Current applications of orcein in histochemistry. A brief review with some new observations concering influence of dye batch variation and ageing of dye solutions on staining. Biotech Histochem.

[17] Friedberg SH, Goldstein DJ (1969). Thermodynamics of orcein staining of elastic fibres. Histochem J.

[18] Dineshshankar J, Ganapathy N, Yoithapprabhunath TR (2019). Morphological analysis of elastic fibres in various grades of oral squamous cell carcinoma and epithelial dysplasia using Verhoeff–Van Gieson stain. Rambam Maimonides Med J.

[19] John RE, Murthy S (2016). Morphological analysis of collagen and elastic fibres in oral squamous cell carcinoma using special stains and comparison with Broder’s and Bryne’s grading systems. Indian J Dent Res.

[20] Broders AC (1941). The microscopic grading of cancer. Surg Clin North Am.

[21] Rajendran R, Sundharam B (2006). Shafers Text Book of Oral Pathology.

[22] Almine JF, Wise SG, Weiss AS (2012). Elastin signalling in wound repair. Birth Defects Res Part C Embryo Today Rev.

[23] Tosios KI, Economou I, Vasilopoulos NN (2010). Elastofibromatous changes and hyperelastosis of the oral mucosa. Head Neck Pathol.

[24] Jamadar S, Narayan T, Shreedhar B (2014). Comparative study of various grading systems in oral squamous cell carcinoma and their value in predicting lymph node metastasis. Indian J Dent Res.

[25] Kardam P, Mehendiratta M, Rehani S (2016). Stromal fibres in oral squamous cell carcinoma: a possible new prognostic indicator?. J Oral Maxillofac Pathol.

[26] Tlsty TD, Coussens LM (2006). Tumour stroma and regulation of cancer development. Annu Rev Pathol.

[27] Peltanova B, Raudenska M, Masarik M (2019). Effect of tumour microenvironment on pathogenesis of the head and neck squamous cell carcinoma: a systematic review.

[28] Kalluri R (2016). The biology and function of fibroblasts in cancer. Nat Rev Cancer.

[29] Özdemir BC, Pentcheva-Hoang T, Carstens JL (2014). Depletion of carcinoma-associated fibroblasts and fibrosis induces immunosuppression and accelerates pancreas cancer with diminished survival. Cancer Cell.

[30] Kalluri R (2003). Basement membranes: structure, assembly and role in tumour angiogenesis. Nat Rev Cancer.

[31] Erler JT, Weaver VM (2009). Three-dimensional context regulation of metastasis. Clin Exp Metastasis.

[32] Spill F, Reynolds DS, Kamm RD (2016). Impact of the physical microenvironment on tumour progression and metastasis. Curr Opin Biotechnol.

[33] Lühr JJ, Alex N, Amon L (2020). Maturation of monocyte-derived DCs leads to increased cellular stiffness, higher membrane fluidity, and changed lipid composition. Front Immunol.

[34] Lu P, Weaver VM, Werb Z (2012). The extracellular matrix: a dynamic niche in cancer progression. J Cell Biol.

[35] Hanahan D, Weinberg RA (2011). Hallmarks of cancer tumour progression only certain paths are available to tumours as they acquire hallmarks.

[36] Erez N, Truitt M, Olson P (2010). Article cancer-associated fibroblasts are activated in incipient neoplasia to orchestrate tumour-promoting inflammation in an NF- k B-dependent manner. Cancer Cell.

[37] Hanahan D, Weinberg RA (2000). The hallmarks of cancer review douglas. Cell.

[38] Hanahan D, Coussens LM (2012). Accessories to the crime: functions of cells recruited to the tumour microenvironment. Cancer Cell.

[39] Pickup MW, Mouw JK, Weaver VM (2014). The extracellular matrix modulates the hallmarks of cancer. EMBO Rep.

[40] Lisa M, Coussens ZW (2002). Inflammation and cancer. Nature.

[41] Mouw JK, Ou G, Weaver VM (2014). Extracellular matrix assembly: a multiscale deconstruction. Nat Rev Mol Cell Biol.

[42] Monteran L, Erez N (2019). The dark side of fibroblasts: cancer-associated fibroblasts as mediators of immunosuppression in the tumour microenvironment. Front Immunol.

[43] Qian X, Xiao F, Chen YY (2021). Computerised assessment of the tumour-stromal ratio and proposal of a novel nomogram for predicting survival in invasive breast cancer. J Cancer.

[44] Khalid A, Siddiqui S, Faizi N (2019). Role of stromal myofibroblasts in the progression of oral lesions from dysplasia to invasive carcinoma. Indian J Med Paediatr Oncol.

[45] Ushiki T, Murakumo M (1991). Scanning electron microscopic studies of tissue elastin components exposed by a KOH-collagenase or simple KOH digestion method. Arch Histol Cytol.

[46] Rajendran R, Sivapathasundharam BE (2006). Shafer, Hine L. Shafer’s Textbook of Oral Pathology.

[47] Unna PG (1890). U¨ ber die Taenzer’sche Fa¨rbung des elastischen Gewebes. Monatsh Prakt Dermatol.

[48] Weigert C, Workman C (1892). On the staining of the medullary sheath of nerve fibres. Glasgow Med J.

[49] Verhoeff FH (1908). Some new staining methods of wide applicability. Including a rapid differential stain for elastic tissue. JAMA.

[50] Gieson IV (1889). Laboratory notes of technical methods for the nervous system. New York Med J.

[51] Russell HK (1972). A modification of Movat’s pentachrome stain. Arch Pathol.

[52] Masson CLP (1929). Some histological methods. Trichrome staining and their preliminary technique. J Tech Methods.

[53] Gömöri G (2000). Gomori ’ s Aldehyde Fuchsin.

[54] Shikata T, Uzawa T, Yoshiwara N (1974). Staining methods of Australian antigen in paraffin sections- detection of cytoplasmic inclusion bodies. Japneses J Exp Med.

[55] Brissie RM, Spicer SS, Thompson NT (1975). The variable fine structure of elastin visualised with verhoeff’s iron hematoxylin. Anat Rec.

[56] Churukian CJ, Schenk EA (1976). Iron gallein elastic method – a substitute for Verhoeff’s elastic tissue stain. Stain Technol.

[57] Krobock E, Rahbari H, Mehregan AH (1978). Acid orcein and giemsa stain: modification of a valuable stain for dermatologic specimens. J Cutan Pathol.

[58] Singh R, Gorton AWP (1989). Orcein-alcian blue staining: a new technique for demonstrating acid mucins in gastrointestinal epithelium. J Clin Pathol.

[59] Chalvardjian A, Lee S (1986). HOPS: a tetrachrome stain useful in dermatopathology. Am J Dermatopathol.

[60] Steven P, Paulsen F, Tillmann B (2000). Orcein-picroindigocarmine – a new multiple stain. Arch Histol Cytol.

[61] Senba M (1984). Histologic staining methods for hepatitis b surface antigen (HBS AG) and interpretation of dye reactions in vitro. Acta Histochemica Et Cytochemica.

[62] Salaspuro M, Sipponen P (1976). Demonstration of an intracellular copper binding protein by orcein staining in long standing cholestatic liver diseases. Gut.

[63] Nakamura H, Kanai C, Mizuhira V (1977). An electron stain for elastic fibres using orcein. J Histochem Cytochem.

[64] Scheuer PJ, Maggi G (1980). Hepatic fibrosis and collapse: histological distinction by orcein staining. Histopathology.

[65] Shousha S, Boxer GM (1981). Orcein as a mucin stain for gastrointestinal tissue sections. J Clin Pathol.

[66] Dawson JF, Brochier J, Schmitt D (1984). Elastic fibres: histological correlation with orcein and a new monoclonal antibody. Br J Dermatol.

[67] Pieraggi M, Nejjar I, Julian M (1986). Staining of elastic tissue by Verhoeff’s iron hematoxylin. Ann Pathol.

[68] Kim JH Comparative study on orcein and victoria blue staining for the demonstration of HBsAg in human liver tissue specimens. Korean J Med Technol.

